# Clinical Importance of Epstein–Barr Virus-Associated Gastric Cancer

**DOI:** 10.3390/cancers10060167

**Published:** 2018-05-29

**Authors:** Jun Nishikawa, Hisashi Iizasa, Hironori Yoshiyama, Kanami Shimokuri, Yuki Kobayashi, Sho Sasaki, Munetaka Nakamura, Hideo Yanai, Kohei Sakai, Yutaka Suehiro, Takahiro Yamasaki, Isao Sakaida

**Affiliations:** 1 Department of Laboratory Science, Yamaguchi University Graduate School of Medicine, 1-1-1 Minami-Kogushi, Ube, Yamaguchi 755-8505, Japan; g006up@yamaguchi-u.ac.jp (K.Sh.); kobadon@yamaguchi-u.ac.jp (Y.K.); 2 Department of Microbiology, Shimane University Faculty of Medicine, 89-1 Enyacho, Izumo, Shimane 693-8501, Japan; iizasah@med.shimane-u.ac.jp (H.I.); yosiyama@med.shimane-u.ac.jp (H.Ya.); 3 Department of Gastroenterology and Hepatology, Yamaguchi University Graduate School of Medicine, 1-1-1 Minami-Kogushi, Ube, Yamaguchi 755-8505, Japan; sho.ssk.nv21@gmail.com (S.S.); munemune84@hotmail.com (M.N.); sakaida@yamaguchi-u.ac.jp (I.S.); 4 Department of Clinical Research, National Hospital Organization Kanmon Medical Center, 1-1 Sotoura, Chofu, Shimonoseki, Yamaguchi 752-8510, Japan; yanaih@kanmon-mc2.hosp.go.jp; 5 Department of Oncology and Laboratory Medicine, Yamaguchi University Graduate School of Medicine, 1-1-1 Minami-Kogushi, Ube, Yamaguchi 755-8505, Japan; sakaik@yamaguchi-u.ac.jp (K.Sa.); ysuehiro@yamaguchi-u.ac.jp (Y.S.); t.yama@yamaguchi-u.ac.jp (T.Y.)

**Keywords:** Epstein–Barr virus, gastric carcinoma, DNA methylation, programed cell death 1 (PD-1), programmed cell death ligand 1 (PD-L1), immune checkpoint inhibitor, endoscopic mucosal resection (EMR), endoscopic submucosal dissection (ESD)

## Abstract

Epstein–Barr virus-associated gastric carcinoma (EBVaGC) is the most common malignancy caused by EBV infection. EBVaGC has definite histological characteristics similar to gastric carcinoma with lymphoid stroma. Clinically, EBVaGC has a significantly low frequency of lymph node metastasis compared with EBV-negative gastric cancer, resulting in a better prognosis. The Cancer Genome Atlas of gastric adenocarcinomas proposed a molecular classification divided into four molecular subtypes: (1) EBVaGC; (2) microsatellite instability; (3) chromosomal instability; and (4) genomically stable tumors. EBVaGC harbors a DNA methylation phenotype, PD-L1 and PD-L2 overexpression, and frequent alterations in the PIK3CA gene. We review clinical importance of EBVaGC and discuss novel therapeutic applications for EBVaGC.

## 1. Introduction

Epstein–Barr virus (EBV) is a ubiquitous human herpes virus that was originally discovered in Burkitt lymphoma. EBV can transform primary B lymphocytes in vitro and is associated with nasopharyngeal carcinoma, Hodgkin lymphoma, natural killer (NK)/T lymphoma, and post-transplant lymphoproliferative disorders [1,2]. Gastric carcinoma is the fourth most frequent cancer in the world and is the second leading cause of cancer deaths. More than 95,000 new patients are diagnosed each year, and there were 72,000 gastric cancer-related deaths worldwide in 2012 [3]. About 9% of gastric cancers have been identified as EBV-positive [4]. Thus, EBV-associated gastric carcinoma (EBVaGC) is the most common cancer among EBV-related malignancies. In the Cancer Genome Atlas (TCGA), gastric cancer is classified according to its molecular biology into EBVaGC, microsatellite unstable tumors, genomically stable tumors, and chromosomally unstable tumors [5]. We review the clinicopathologic and molecular features of EBVaGC and discuss novel therapeutic applications for EBVaGC.

## 2. Definition

**Figure 1:**
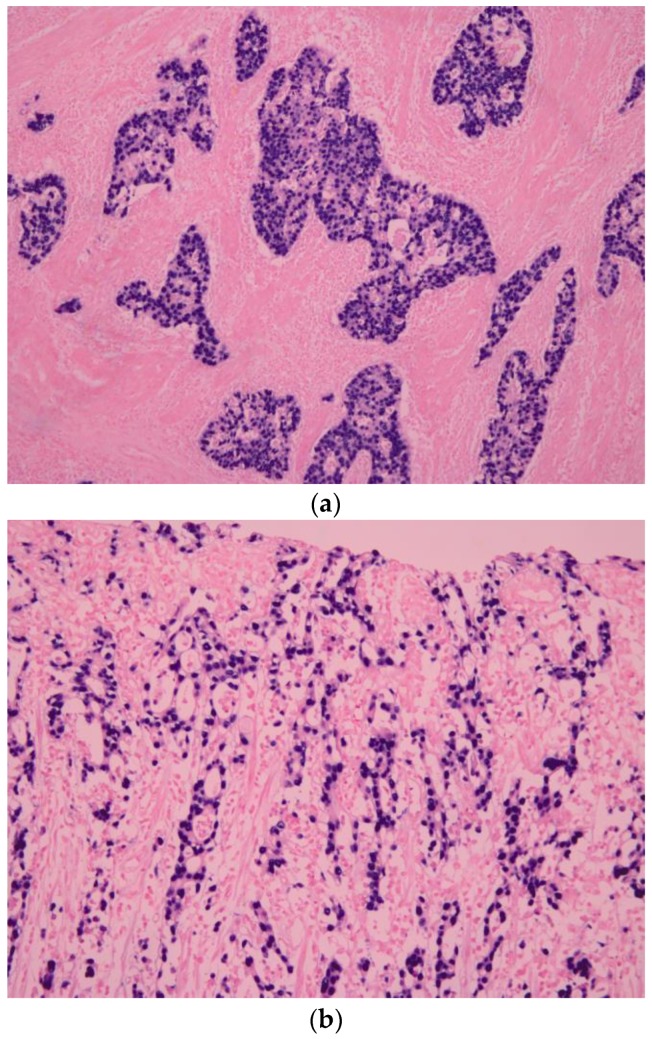
Histologic characteristic of Epstein–Barr virus-associated gastric carcinoma (EBVaGC). (**a**) EBV-encoded small ribonucleic acid (EBER1) in situ hybridization shows positive nuclei in the carcinoma cells, which are surrounded by infiltrating lymphocytes (×100); (**b**) Histologic characteristic of EBVaGC. The “lacy pattern” is composed of irregularly anastomosing tubules and moderate to dense lymphocytic infiltration (×100).

In 1990, Burke et al. reported EBV-positive gastric carcinoma by PCR [6]. In the early 1990s, various studies showed that EBVaGC comprises about 10% of all gastric cancers worldwide by using in situ hybridization (ISH) targeting EBV-encoded small RNA 1 (EBER1) [7,8]. EBER1 is abundant (10 million copies per cell) in latently EBV-infected cells. EBER1 ISH is commonly used to assess EBV infection in histopathologic samples. Typically, signals of EBER1 are detected in the nuclei of almost all carcinoma cells in EBVaGC (Figure 1). Imai et al. [9] and Fukayama et al. [10] reported that EBVaGC resulted from the monoclonal proliferation of EBV-infected cells.

## 3. Pathologic Features

EBVaGC has definite histological relevance to carcinoma with lymphoid stroma (CLS) [11,12,13], which was originally reported by Watanabe et al. [14]. CLS is a poorly differentiated adenocarcinoma with diffuse and intense lymphocyte infiltration and resembles EBV-positive nasopharyngeal carcinoma (Figure 1a). The incidence of CLS was reported to be 3–4% of all gastric cancers. More than 80% of CLS is infected with EBV [11,12,13], whereas EBV was observed in 5–10% of ordinary-type gastric cancers that show features of moderately or poorly differentiated adenocarcinoma with various amount of lymphocytic infiltration.

It is known that *Helicobacter pylori (H. pylori)* is causally associated with gastric cancer development and is a pathogenic factor of chronic gastritis and intestinal metaplasia [15]. Several studies reported the preventive effect of *H. pylori* eradication on gastric cancer development [16,17]. We have also reported that EBVaGC is derived from gastric mucosa with chronic inflammation induced by *H. pylori* infection [18]. Although EBVaGC predominantly localizes in the middle or upper stomach, the background mucosa of EBVaGC is accompanied by mucosal atrophy and intestinal metaplasia [18]. It is uncertain whether *H. pylori* eradication has a preventive effect on the development of EBVaGC.

When cases of CLS are observed, EBER ISH should be performed to aid in the diagnosis. Tokunaga et al. reported that early-stage EBVaGC shows a characteristic histology called a “lacy pattern” that is composed of irregularly anastomosing tubules and moderate to dense lymphocytic infiltration (Figure 1b) [19]. Other pathologic characteristics were also reported; however, EBER1 ISH is necessary to prove EBV infection in tumor cells.

## 4. Clinical Features

Almost all studies show male predominance of EBVaGC [4]. Camargo et al. reported that the association of smoking with gastric cancer is stronger for EBVaGCs than for control cases [20].

**Figure 2:**
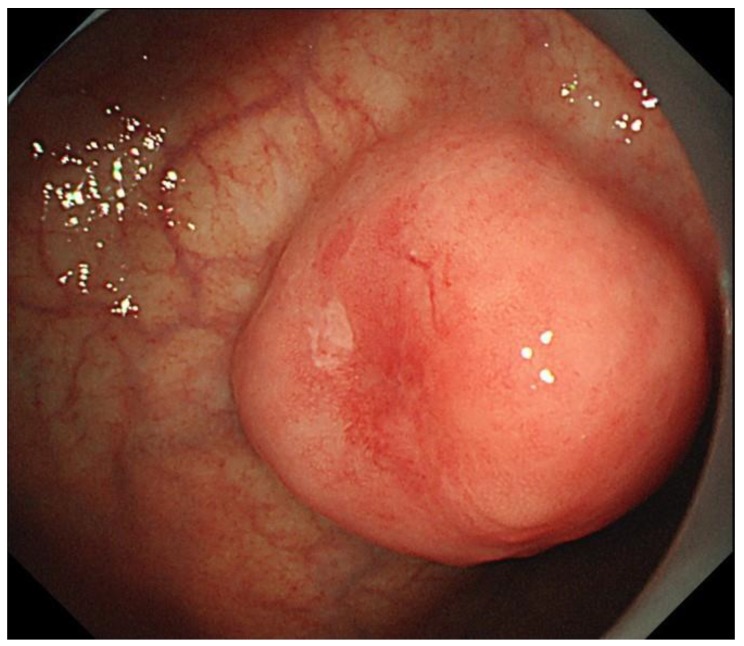
Endoscopic images of an EBV-associated gastric carcinoma. Submucosal tumor-like morphology is one of the features of EBVaGC.

We reported endoscopic and endosonographic features of EBVaGC. EBVaGC predominantly localizes in the middle or upper stomach and appears as superficially depressed or ulcerated lesions [21]. It is considered that submucosal tumor-like morphology is one of the features of CLS. Although the majority of gastric CLS overlaps with EBVaGC, the submucosal tumor-like morphology may be helpful in discovering EBVaGC (Figure 2) [21]. Endosonography revealed a hypoechoic mass in the submucosal layer reflecting nodules, which is composed of tumor cells and infiltrating lymphocytes [22].

Gastric remnant cancer can arise after distal gastrectomy for gastroduodenal ulcers accompanied by bleeding or perforation. It is frequently (25% to 41.2%) associated with EBV infection [23,24]. A high prevalence of EBVaGC was reported in synchronous and metachronous gastric cancers [25,26]; however, this prevalence is not evident in meta-analyses of the characteristics of EBVaGC. Background mucosa in which EBVaGC had once been present might be at high risk for the redevelopment of EBVaGC.

A clinicopathological study showed that the frequency of lymph node metastasis (LNM) is significantly low in EBVaGCs compared with EBV-negative controls [27]. The rates of LNM in mucosa-confined and submucosal early gastric cancers are 2.2–4.6% and 14.0–23.6%, respectively [28,29,30,31,32,33]. The frequency of LNM is even lower in early EBVaGC. Tokunaga et al. reported that early-stage EBVaGC did not have LNM even in submucosally invading gastric cancers [34]. Park et al. showed that EBV negativity was an independent risk factor for LNM in submucosal early gastric cancers [35]. A recent meta-analysis revealed the infrequent tendency of EBVaGC toward LNM. After disease stage and prognostic factors were adjusted for, EBV positivity was associated with lower mortality [36].

## 5. Molecular Features of EBVaGC by TCGA Project

TCGA of gastric adenocarcinomas [5] divided gastric cancer into four molecular subtypes: (1) EBVaGC; (2) microsatellite instability; (3) chromosomal instability; and (4) genomically stable tumors. In TCGA project, 26 tumor samples of EBVaGCs were analyzed. The characteristics of EBVaGC are reported to include the harboring of recurrent PIK3CA mutations, DNA hypermethylation, amplification of JAK2, and overexpression of both PD-L1 and PD-L2 [5]. Recently, many investigators have reported PD-L1 overexpression in EBVaGC [37,38,39,40,41]. Crescenzi et al. reported that PD-L1 is expressed in gastric cancer cells, whereas PD-1 is expressed in infiltrating lymphocytes [42]. It seems that EBVaGC evades immunity from immunological recognition by T lymphocytes via the PD-1/PD-L1 pathway.

DNA methylation plays an important role in the development and progression of gastric cancer [43]. Methylation of both viral and host DNA is important for the development of EBVaGC. Viral DNA methylation controls the expression of EBV latent and lytic genes. Methylation of host cell DNA inactivated tumor suppressor genes and tumor-associated antigens [44]. Methylation frequencies of several tumor-related genes and DNA loci were reported to be significantly higher in EBVaGC [45,46,47,48,49,50]. The molecular mechanism that induces DNA methylation in EBVaGC has also been studied. Hino et al. showed that EBV latent membrane protein 2A (LMP2A) induces DNA methyltransferase 1 transcription via the phosphorylation of STAT3 [51]. Namba-Fukuyo et al. reported that TET2 downregulation was crucial in inducing DNA methylation in EBVaGC. Although the TET family genes encoded DNA demethylase, TET2 was downregulated by EBV transcripts such as BARF0 and LMP2A and TET2 targeting miRNAs [52]. Matsusaka et al. showed that when EBV was infected into MKN7 gastric cancer cells and the GES1 non-neoplastic gastric epithelial cell line, DNA methylation was induced in these cells [53,54].

The carcinogenic interaction between *H. pylori* and EBV was recently reported. Tyrosine-phosphorylated cytotoxin-associated gene A (*Cag A*) binds to the tyrosine phosphatase SHP2 and deregulates the phosphatase activity, which has been considered to play an important role in gastric carcinogenesis [55,56]. SHP2 homologue SHP1 also interacts with Cag A, but SHP1 dampens the oncogenic function of Cag A. Saju et al. reported that EBV infection can induce promoter methylation of SHP1 and downregulate the expression of SHP1, which might increase the oncogenic function caused by the interaction of Cag A and SHP2 [57].

## 6. Treatment of EBVaGC

Most EBVaGCs are surgically resected, and better prognosis in these patients has already been reported [36].

### 6.1. Treatment for Early-Stage EBVaGC

Early gastric cancer is defined as cancer confined to the gastric mucosa or submucosa, and the 5-year survival rate of early gastric cancer exceeds 90% in Japan. Because endoscopic examination is highly advanced in Japan, more than 50% of gastric cancer cases are early detections. Endoscopic mucosal resection (EMR) was designed as a local treatment for early gastric cancers appearing with no LNM [58]. The guideline criteria for EMR according to the Japanese Gastric Cancer Association is mucosal cancer ≤20 mm in size without ulcerative findings [59]. When lesions fit these criteria, EMR achieves results equal to those of surgical resection [60,61]. Endoscopic submucosal dissection (ESD) is a novel endoscopic technique that enables the resection of larger lesions en bloc. Gotoda et al. analyzed 5265 early gastric cancer patients who underwent gastrectomy with lymph node dissection. They provided important information on the risks of LNM and proposed expanded criteria for endoscopic resection: (1) mucosal cancer without ulcer findings, irrespective of tumor size; (2) mucosal cancer with an ulcer ≤3 cm in diameter; and (3) minimal (≤500 μm from the muscularis mucosa) submucosal invasive cancer ≤3 cm in size [28]. Use of the ESD method has achieved curative resections in both the guideline and the expanded-criteria lesions.

EBVaGC has a significantly low frequency of LNM compared with EBV-negative gastric cancer, especially in the early stage [34,35,36]. The criteria of endoscopic resection have been carefully expanded. In the future, EBV-associated early gastric cancers will become an indication for minimally invasive therapy such as EMR and ESD because LNM in submucosal invasive cancer with EBV infection is low.

### 6.2. Treatment for Advanced-Stage EBVaGC

#### 6.2.1. DNA Methylation Inhibitors

**Figure 3:**
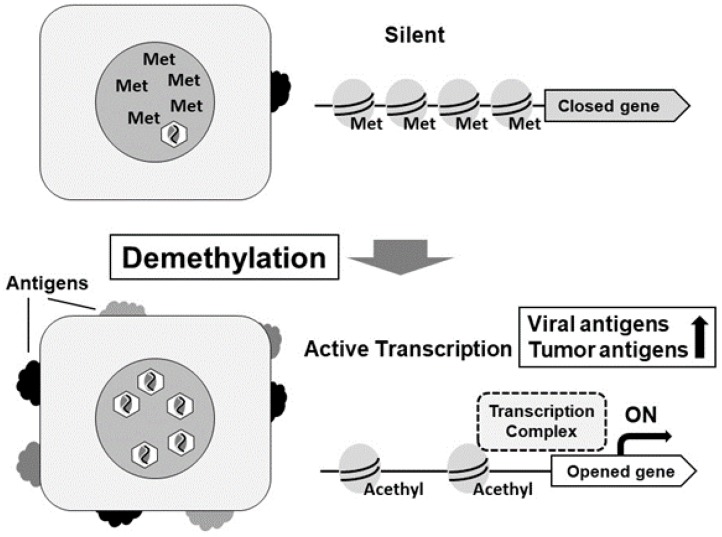
Treatment by DNA demethylating agents for EBVaGC.

The therapeutic application of DNA demethylating agents for EBVaGC is an attractive approach. Because demethylation agents induce lytic EBV infection in latently EBV-infected cells followed by apoptotic cell death, the demethylating agents may lead to the lysis of cancer cells. [47,48]. We investigated the anticancer effects of 5-aza-2′-deoxycytidine (decitabine) against EBVaGC cell lines. Decitabine induced G2/M arrest, apoptosis, and the expression of E-cadherin in the cells. The promoters of tumor suppressor genes were demethylated, and their expression was upregulated by decitabine treatment [62]. These facts strongly support the possible application of demethylating agents in the medical treatment of EBV-associated gastric cancer (Figure 3).

DNA methylation inhibitors 5-azacytidine and decitabine were approved by the US Food and Drug Administration in 2004 and 2006, respectively, for the treatment of patients with myelodysplastic syndromes. Decitabine was efficacious in solid tumors, including lung cancer, esophageal cancer, and pleural mesothelioma [63]. Epigenetic agents showed antitumor activity against solid tumors by the single or combinational use with standard anticancer treatment. A phase 1 study was conducted to identify a tolerable dose of 5-azacitidine prior to neoadjuvant chemotherapy in patients with locally advanced esophageal/gastric adenocarcinoma. Neoadjuvant administration together with 5-azacitidine with chemotherapy was tolerated with significant clinical and epigenetic responses. Several tumor suppressor genes were demethylated and expressed in the tumors resected surgically [64].

The efficacy of decitabine is limited because of its instability in vivo [65]. Nanoparticle-based drug delivery systems have rapidly developed in the field of cancer therapeutics. Many reports have confirmed the superiority of nanoparticles, such as decitabine-loaded nanogels [66]. Decitabine in nanogel sustains DNMT1 depletion and makes cancer cells stay in G2/M arrest. Nanoparticle-based drug delivery systems could potentially be explored to treat solid tumors as well [67].

#### 6.2.2. Immune Checkpoint Inhibitor

Treatment by blocking PD-1/PD-L1, which is an immune checkpoint molecule for the immunoregulatory system, currently shows good prognosis in various cancers. The anti-PD-1 antibody nivolumab showed therapeutic effects in malignant melanoma, non-small cell lung cancer, renal cell carcinoma, and Hodgkin lymphoma [68,69,70,71,72,73]. Recently, patients with advanced or recurrent gastric cancer who could not undergo standard therapy due to their physical condition or treatment intolerance have been treated with nivolumab. These patients showed significantly prolonged overall survival in comparison with those receiving a placebo [74].

**Figure 4:**
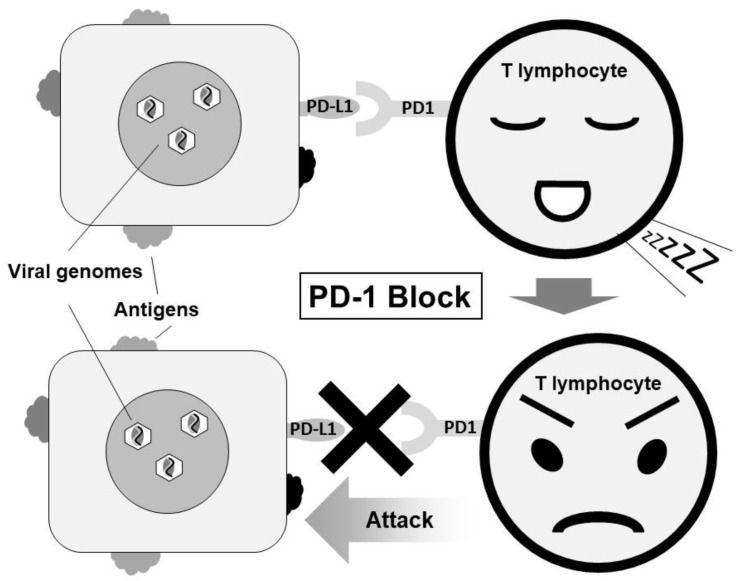
Treatment by blocking PD-1/PD-L1 for EBVaGC.

It is important to search for biomarkers that predict the effects of anti-PD-1/PD-L1 antibody therapy. Previous studies reported that tumors with high expression levels of PD-L1 and tumors with lymphocytic infiltration in the stroma are more likely to respond to anti-PD-1 antibody therapy [74,75]. The expression of PD-L1 is common in EBVaGC and EBV-associated post-transplant lymphoproliferative disorder, NK/T lymphoma, Hodgkin lymphoma, and nasopharyngeal carcinoma [76,77]. The constitutive expression of PD-L1 might be involved in the development of these EBV-related tumors. It is reported that carcinomas sensitive to immune checkpoint inhibitors have numerous genetic mutations by expressing diverse neoantigens. In particular, EBVaGC expresses EBV latent and lytic genes, which may also act as neoantigens (Figure 4).

There is a strong urge to identify a biomarker predicting the response to checkpoint blockades because EBV-associated cancers, especially EBVaGC, could be therapeutic targets [78]. The new marker may also be applied to treat virus-related malignancy by developing novel immunotherapy.

### 6.3. Potential Therapies for EBVaGC

#### 6.3.1. Proteasome Inhibitors

Proteasome is an enzyme complex localized in the cytoplasm and the nucleus that maintains the intracellular environment by degrading and removing ubiquitinated proteins. EBV infection protects infected cells from immunological attack and apoptosis by accelerating degradation of host proteins by proteasome. BDLF3 assists in evading immune recognition because this EBV protein induces ubiquitination of both major histocompatibility complex (MHC)-I and MHC-II molecules [79]. BZLF1 is an inducer of EBV lytic infection, which forms a complex with the apoptosis inducer p53, but is subject to ubiquitination [80]. Therefore, inhibition of proteasomal function in EBV infected cells induces viral lytic infection and apoptosis of infected cells. Single or combinational use of the proteasome inhibitor bortezomib with radiation therapy suppressed tumor formation in mice transplanted with primary EBV-associated gastric cancer cells [81]. However, therapeutic application of bortezomib is limited because this drug has serious side effects such as thrombocytopenia.

#### 6.3.2. Infusion of EBV-Specific Cytotoxic T Cells

In immunologically competent persons who are infected with EBV, most of the EBV-antigen expressing cells have been removed from the body by EBV-specific cytotoxic T cells (EBV-CTL). Therefore, EBV-CTL therapy can be administered to the EBV-infected cancer. Such an attempt was applied to treat lymphoproliferative disorders caused after transplantation because EBV antigens are highly expressed on the proliferating B cells [82]. Moreover, a recent study extended EBV-CTL therapy to EBV-positive epithelial cell tumors. Straathof and colleagues reported that four of ten nasopharyngeal cancer patients who received EBV-CTL went into remission, and 2 of the 6 refractory patients exhibited immune responses [83]. However, the efficacy of EBV-CTL is influenced by the combination of MHC genes and antigen epitopes. It is hard to expect a therapeutic effect of EBV-CTL against EBV-associated gastric cancer because EBV-associated gastric cancer cells exhibit type I latency, which expresses EBNA1 with low antigenicity or LMP2A only in some of the cells.

#### 6.3.3. Histone Deacetylase (HDAC) Inhibitors

Histone modification is known to control lytic infection of EBV [84]. Hui et al. reported that HDAC inhibitors induced lytic infection in latently EBV-infected gastric cancer cell lines [85]. EBV is not sensitive to ganciclovir because the viral thymidine kinase is not expressed during latent infection. However, Hui et al. succeeded in the targeted killing of infected cells by combinational use of ganciclovir and the HDAC inhibitor romidepsin by inducing lytic replicative infection [85].

#### 6.3.4. Indoleamine 2,3-Dioxygenase (IDO) Inhibitors

Depletion of tryptophan inhibits the expression of NK cell receptor NKG2D, which results in the suppression of T-cell proliferation [86]. Expression of IDO, a tryptophan metabolizing enzyme, is elevated in EBV-infected cells. IDO induces immunological evasion of infected cells from NK cell recognition [87]. Thus, IDO inhibitors can be applied to remove EBV-infected cells.

#### 6.3.5. Small-Molecule EBNA1 Inhibitors

EBNA1 plays an important role in establishing persistent infection of EBV because the molecule binds and cross-links viral genomes and human genomes. Thus, EBNA1 is expressed in any of EBV-associated cancer cells. Thompson and colleagues screened EBNA1 inhibitors and found 3 structural EBNA1 mimics from 14,000 substances [88]. Treatment of Raji cells with EBNA1 inhibitors reduced the EBV copy number of the cells in a dose-dependent manner. Similar inhibitors discovered by in silico virtual screening are regarded as attractive candidates for EBV eliminators [89].

#### 6.3.6. EBV Vaccine

Although the development of live vaccine is facing difficulties, development of component EBV vaccines for preventive and therapeutic use has been continuing. The gp350 glycoprotein is used for most vaccines as an antigen. Moreover, EBNA1 and LMP2A have also been used as antigens [90]. In addition to the development of component vaccines, another approach is also being investigated. AMMOM1 is a monoclonal antibody of EBV gH/gL, which inhibits viral infection to B cells and epithelial cells [91]. By utilizing the epitope recognized by the antibody, it might be possible to develop a novel type of EBV vaccine.

## 7. Summary

We reviewed the clinicopathologic and molecular features of EBVaGC. Due to the distinct characteristics of EBVaGC, minimally invasive surgery such as endoscopic resection could be indicated for EBVaGC in the early stage. DNA methylation inhibitor and immune checkpoint inhibitor treatment could be suitable targets for EBVaGC in the advanced stage.
